# Error in Results

**DOI:** 10.1001/jamanetworkopen.2020.20927

**Published:** 2020-09-10

In the Original Investigation titled “Changes in the Number of US Patients With Newly Identified Cancer Before and During the Coronavirus Disease 2019 (COVID-19) Pandemic,”^1^ published August 4, 2020, there were errors in the Figure and Results sections. The percentage of patients who were included in the COVID-19 period was 7.2%. The denominators given in the distribution of women with pancreatic cancer has been changed to 16 248 and 1546 to account for 21 patients who did not have gender information. In the Figure, an additional column was added to represent data on esophageal cancer, and the dates of the weeks in the periods examined were adjusted by 1 day. This article has been corrected.^1^
